# Enterovirus 71 related severe hand, foot and mouth disease outbreaks in South-East Asia: current situation and ongoing challenges

**DOI:** 10.1136/jech-2014-203836

**Published:** 2014-03-20

**Authors:** Saraswathy Sabanathan, Le Van Tan, Louise Thwaites, Bridget Wills, Phan Tu Qui, H Rogier van Doorn

**Affiliations:** 1 Oxford University Clinical Research Unit, Wellcome Trust Major Overseas Programme, Ho Chi Minh City, Viet Nam; 2 Hospital of Tropical Diseases, Ho Chi Minh City, Viet Nam; 3 Nuffield Department of Medicine, Centre for Tropical Medicine, University of Oxford, Oxford, UK

**Keywords:** Developing Countr, Epidemics, International Hlth

## Introduction

In early 2012, doctors in Cambodia noticed high numbers of infants and young children presenting with a severe and unusual illness. The striking features of the disease were an initial encephalitic presentation followed by a rapidly fatal destructive alveolar pneumonia, alarming experienced clinicians. Between April and July 2012 a total of 78 children were affected, 54 of whom died. Enterovirus 71 (EV71) was identified as the causative organism, possibly aggravated by malnutrition and uncontrolled use of steroids.^1^ EV71 is one of the pathogens associated with hand, foot and mouth disease (HFMD). It was responsible for large HFMD outbreaks in Taiwan (1.5 million cases) and Malaysia (Sarawak, 2628 cases) in the late 1990s. In 2008 and 2011 large outbreaks were also described in China (490 000 cases) and Vietnam (110 000 cases).^2^ The recent Cambodian outbreak further demonstrates the emergence and spread of serious EV71 related disease in the South-East Asian region over the last two decades.

## Disease, pathogen, epidemiology

The clinical syndrome of fever with oral ulcers and exanthema on the hands and feet of young children was first observed in 1957 in New Zealand. The term ‘Hand, foot and mouth disease’ was first used the following year.^3^ HFMD is mostly caused by EVs belonging to the species *Enterovirus A* (consisting of Coxsackie viruses A 2–8, 10, 12, 14, 16 and EVs 71, 76 and 89–92). EV71 is thought to have evolved from Coxsackie virus A16 around 1940,^4^ subsequently diverging into three lineages, A, B and C. Lineages B and C are further divided into five sublineages, with B4, B5, C4 and C5 the dominant sublineages identified in recent years in South-East Asia. Although genetically different, all lineages and sublineages represent one serotype of EV71.

HFMD is typically a benign self-limiting illness observed among young children and infants. Outbreaks are often associated with day care centres, nurseries and primary schools. Vesicular lesions usually occur on the palms of the hands and soles of the feet, but all parts of the limbs, including groins and buttocks may be affected. Oral ulceration is usually present, and there is clinical overlap with herpangina, an illness characterised by high fever, sore throat and oral ulceration that also affects young children. Herpangina is caused by a number of EV species.

Prior to its association with HFMD, EV71 had already been detected in a child with encephalitis in 1969 in California. The first associations between EV71 and a more unusual form of HFMD with high numbers of complications were seen in Japan in 1973 and 1978.^5^ Large outbreaks of EV71 associated encephalitis or poliomyelitis-like illness with high mortality were also reported from Europe, North America and Australia during the 1970s.^6^ In the 1998 HFMD Taiwan outbreak, the frequency of EV71 detection increased as the severity and lethality of complications increased.^7^ Currently, EV71 associated HFMD is considered to be endemic in several South-East Asian countries and as an emerging infection in others, with serious concerns regarding the potential for spread beyond the region. In countries such as Japan and Malaysia with a long history of EV71 related disease, a cyclical pattern of outbreaks every 2–3 years is typically observed. This pattern is assumed to relate to the build-up of a large population of susceptible children every few years sufficient to sustain transmission. In China, where outbreaks have been more recent annual peaks of HFMD are observed.^8^

In addition, serological evidence suggests that by the age of 10 years, healthy children in North America and Vietnam have been exposed to many EVs including EV71.^9^ ^10^

### Pathogenesis, clinical features and outcomes

Humans are the only known hosts for HFMD associated EV infections. EVs are non-enveloped viruses and are therefore highly resistant to environmental conditions, and also to mild disinfectants. Transmission is thought to occur primarily through the faecal-oral route, although virus has also been detected in respiratory secretions and in skin lesions. Incubation time is reported to be between 3 days and 6 days, that is, shorter than for poliomyelitis, which has a typical incubation period of 9–12 days. Initial viral replication is presumed to occur in the lymphoid tissues of the oropharyngeal cavity (tonsils) and small bowel (Peyer's patches), giving rise to a mild viraemia. Most infections are successfully controlled at this point and remain asymptomatic. Further dissemination of EVs to the reticuloendothelial system (liver, spleen, bone marrow and lymph nodes), skin and mucous membranes coincides with the onset of clinical symptoms. EV71 can invade central parts of the brain, possibly by retrograde axonal transport. Patients with central nervous system involvement typically present with features of brainstem encephalitis, including myoclonus and autonomic dysregulation (hypertension, tachycardia). A small proportion of these patients may progress to develop cardiopulmonary failure, which may be fatal. The mechanisms underlying the cardiopulmonary failure are thought to be neurogenic in origin, with disproportionate sympathetic stimulation and catecholamine secretion directly affecting the cardiac muscle and raising pulmonary pressures. MRI of the brain suggests typical involvement of the medulla oblongata, pons, midbrain and spinal cord, that resolves within 2 months in most cases.^6^ A prospective study of 730 children admitted with HFMD in Sarawak found high fever, fever for more than 3 days and lethargy as risk factors for central nervous system involvement.^11^ However the positive predictive values for these risk factors were relatively low, resulting in high numbers of hospitalisations for relatively mild disease.

Chinese surveillance data from 2008 to 2012 of over seven million children with HFMD identified the highest incidence to be in children aged 12–23 months. Children aged less than 6 months had the highest risk for severe and fatal disease, with the risk declining with increasing age. Of the 1.1% that had neurological or cardiopulmonary complications, 3% died. Overall, the case-fatality rate was 0.03% (n=2457), and 93% of the laboratory-confirmed deaths (n=1737) were associated with EV71.^8^ In contrast, worldwide 385, 71 and 79 patients died from influenza virus A/H5N1, A/H7N9 and Middle East respiratory syndrome-coronavirus, respectively, between 1997 and 2103.^12^ Feeding difficulties, ventilator dependence due to central hypoventilation and persistent limb weakness have been reported as sequelae following severe disease. Attention deficit hyperactivity in school-aged children, and speech delay in children under 2 years have been reported among milder cases.^13^ ^14^ However the long-term effects on the large numbers of children affected by these outbreaks remain unknown.

## Management, public health and prevention

In 2011, WHO published guidelines for clinical diagnosis and management of HFMD.^15^ Individual countries that have experienced large outbreaks (Taiwan, China, Vietnam) have developed their own guidelines. All the guidelines are based primarily on expert opinion with little evidence base. Most describe a staging or grading system with uncomplicated febrile HFMD designated as Grade 1, high or prolonged fever and/or neurological manifestations such as myoclonus, aseptic meningitis or encephalitis designated as Grade 2, while clear evidence of autonomic nervous system dysregulation classifies the patient as having Grade 3 disease, and cardiopulmonary compromise as Grade 4 disease.

Interventions are tailored to the severity of the disease, and consist of antipyretics, sedatives, intravenous immunoglobulin (IVIG), milrinone, haemofiltration and, occasionally, provision of respiratory support. IVIG is widely used, particularly in Vietnam; it is a very costly intervention with potential risks, and currently there is no evidence to support its use. Milrinone has been shown to reduce mortality in severe cases with cardiopulmonary collapse through a small randomised controlled open-label trial.^16^ It is believed that controlling autonomic dysfunction may avoid progression of disease to cardiopulmonary failure. One novel therapy under investigation is intravenous magnesium sulfate. Magnesium sulfate, long used in the management of pre-eclampsia, promotes cardiovascular stability through a combination of vasodilatation and negative chronotropicity. It also inhibits catecholamine release, and may reduce autonomic dysfunction in tetanus.^17^ ^18^ A randomised placebo controlled trial of magnesium sulfate in children with autonomic dysfunction is in progress in Vietnam to address the potential benefit of this cheap, accessible therapy. (Clinicaltrials.gov number: NCT01940250.) The WHO Western Pacific Region Office provides updates on HFMD cases notified through local public health systems in Japan, Singapore, China, Vietnam, The Philippines and Thailand.^2^ Outbreak control measures are targeted at interrupting virus transmission from person to person, as well as through contact with contaminated surfaces (such as toys) or fomites. Therefore health education messages focus on personal hygiene and good sanitation, including recommendations for frequent hand washing, proper disposal of soiled diapers and regular disinfection of soiled surfaces. The transmission of EVs is most efficient in crowded settings, and therefore during outbreaks most countries in the region have adopted social distancing measures, such as closures of childcare facilities and schools and cancellation of public functions involving children. Little systematic research has been done to assess the effectiveness of such measures, but one study from Singapore seemed to show some benefit.^19^ However, the optimal timing for implementation of these measures is unclear—early, as soon as a HFMD is reported, or later following confirmation of EV71 as the causative agent. In addition, the effectiveness of distancing measures, which have substantial socioeconomic implications, is uncertain. If EV71 is like other directly transmissible viruses, such controls may decrease the peak incidence of disease during an outbreak/epidemic, and blunt the burden on the healthcare system, which can be of great value. However, it could thereby also prolong the outbreak and the total numbers would be similar. In addition, school closure could cause relocation to other geographical areas.^6^

## Vaccines

Phase II and III trials of an inactivated EV71 vaccine have been completed in China. The results are hopeful, with a demonstrated good safety profile and vaccine efficacy of 90% against EV71 associated HFMD. The vaccine consists of an inactivated sublineage C4 virus that is currently prevalent. However it is unclear whether the Chinese vaccine can be deployed across the whole region and whether this specific sublineage will also provide long-term protection against other EV71 sublineages, or against related viruses such as Coxsackie virus A16, because the antigenic distances between these viruses remain unknown.^20^^–^22 Malaysia, Taiwan and Singapore are also currently conducting vaccine research.

## Challenges

As yet, EV71 HFMD outbreaks have not spread beyond South-East Asia. It is important to monitor and study this disease and pathogen to evaluate future risk. The role of host genetics and the duration and efficacy of cross protection provided by past infections with any of the closely related human EVs will need to be assessed to evaluate pandemic potential. Vaccine development has progressed but routine use is still distant. Monitoring viral genetic and antigenic evolution, cross protection and waning of immunity will inform vaccine development and implementation. Public health interventions should be based on understanding transmission chains of EVs, and the duration of viral shedding. There is an urgent need to identify early clinical predictors of severe disease. Countries currently affected typically have frail health infrastructures which can easily be overburdened by outbreaks. Costly interventions already implemented have not been clearly evaluated. Severe HFMD occurs at a time of rapid brain development. Concerns over long-tem outcomes have not been prospectively evaluated. If long-term sequelae are present, the economic and social burden of the disease may be higher than that seen during the acute illness.

These challenges are immense. A first step is to establish regional clinical and laboratory networks. This is crucial to harmonise diagnosis, treatment and management protocols across countries and monitor the evolutionary biology of EV71.

Improved evidence-based management of outbreaks in affected regions is essential preparation for potential outbreaks in novel regions. Multicentre intervention trials allow implementation of successful interventions in several countries at once. Studies are underway in Vietnam to assess the efficacy of magnesium sulfate as a therapeutic intervention, to identify genetic and antigenic evolution of the virus, to look at host genetic factors associated with severe disease, and to evaluate clinical predictors of disease progression and neurodevelopmental complications of severe disease. EV71 and HFMD will continue to be a serious public health issue in South-East Asia.
